# Genome-Wide Analysis of the Role of NAC Family in Flower Development and Abiotic Stress Responses in *Cleistogenes songorica*

**DOI:** 10.3390/genes11080927

**Published:** 2020-08-12

**Authors:** Xifang Zong, Qi Yan, Fan Wu, Qian Ma, Jiyu Zhang

**Affiliations:** State Key Laboratory of Grassland Agro-Ecosystems, Key Laboratory of Grassland Livestock Industry Innovation, Ministry of Agriculture and Rural Affairs, College of Pastoral Agriculture Science and Technology, Lanzhou University, Lanzhou 730020, China; zongxf15@lzu.edu.cn (X.Z.); yanq16@lzu.edu.cn (Q.Y.); wuf15@lzu.edu.cn (F.W.); maq18@lzu.edu.cn (Q.M.)

**Keywords:** *Cleistogenes songorica*, NAC, genome-wide, abiotic stress, cleistogamous

## Abstract

Plant-specific NAC (*NAM*, *ATAF*, *CUC*) transcription factor (TF) family plays important roles in biological processes such as plant growth and response to stress. Nevertheless, no information is known about NAC TFs in *Cleistogenes songorica*, a prominent xerophyte desert grass in northwestern China. In this study, 162 *NAC* genes were found from the *Cleistogenes songorica* genome, among which 156 *C. songorica* *NAC* (*CsNAC*) genes (96.3%) were mapped onto 20 chromosomes. The phylogenetic tree constructed by CsNAC and rice NAC TFs can be separated into 14 subfamilies. Syntenic and Ka/Ks analyses showed that *CsNACs* were primarily expanded by genomewide replication events, and purifying selection was the primary force driving the evolution of *CsNAC* family genes. The *CsNAC* gene expression profiles showed that 36 *CsNAC* genes showed differential expression between cleistogamous (CL) and chasmogamous (CH) flowers. One hundred and two *CsNAC* genes showed differential expression under heat, cold, drought, salt and ABA treatment. Twenty-three *CsNAC* genes were commonly differentially expressed both under stress responses and during dimorphic floret development. Gene Ontology (GO) annotation, coexpression network and qRT-PCR tests revealed that these *CsNAC* genes may simultaneously regulate dimorphic floret development and the response to stress. Our results may help to characterize the NAC transcription factors in *C. songorica* and provide new insights into the functional research and application of the NAC family in crop improvement, especially in dimorphic floret plants.

## 1. Introduction

The agricultural yield is affected by environmental factors. Stress incidents pose huge challenges to global agricultural production, causing many losses every year [1]. Plants may suffer from various environmental stresses at different stages of growth [2]. To survive in these severe environments, plants evolve complex genetic mechanisms that regulate gene expression through accurate transcriptional control and precise signal transduction.

Transcription factors (TFs) combine with corresponding *cis*-acting elements to activate or inhibit the expression of their target genes and are crucial regulatory factors in many signaling networks [3]. TFs are involved in the regulation of many biological processes, including cellular morphogenesis, metabolic process, signal transduction and stress response [4]. The NAC (*NAM*, *ATAF* and *CUC2*) family is one of the largest families of plant-specific transcription factors and exists widely in numerous plants [5,6]. The *NAC* gene was firstly reported to be involved in forming shoot apical meristem and primordium in *Petunia hybia* [7]. Generally, the NAC family possesses a highly conserved N-terminal region (NAM domain), and there are at least five subdomains [5]. Furthermore, the relatively divergent C-terminal transcriptional regulation region of NAC TFs can repress or activate the transcription of multiple target genes [8]. In addition, some NAC proteins possess α-helical transmembrane motifs (TMs) at their C-terminus for binding to the plasma membrane [9,10].

The NAC TFs are implicated in plant growth and development processes, including shoot apical meristem formation [7], flower morphogenesis [11], leaf senescence, hormonal signaling [12], stomatal closure [13], plant height [14], cell division and metabolism [15,16], secondary cell wall biosynthesis [17], lateral root formation [18], seed development and germination [19,20], fruit ripening [21] and crop yield and quality [22]. The leaf aging of transgenic *Arabidopsis* overexpressing the *VND-INTERACTING2* (*VNI2;* an NAC transcription factor) gene was delayed, while leaf aging of *VNI2*-deficient mutants was significantly accelerated [23]. Inhibition of the expression of *ZmNAC130* resulted in the reduction in starch in maize [20]. *MdNAC1* was found to be associated with plant height, and overexpression of the gene resulted in a dwarf phenotype in transgenic apple plants. Furthermore, NAC TFs are involved in responding to multiple stresses, such as pathogen immunity [24], salt [25], drought [26], cold [27] and heat [28]. The membrane-bound protein NTL8 induced by salt stress can regulate *Arabidopsis* seed germination through the gibberellin acid (GA) pathway. Germination of T-DNA inserted *ntl8–1* mutant seeds resistant to paclobutrazol (PAC; GA biosynthetic inhibitor) and high salinity [19]. The nucleus-localized protein ONAC066 can activate the expression of *OsDREB2A* (a drought-responsive gene) by combining with the AtJUB1 binding site in the *OsDREB2A* promoter. Transgenic rice lines overexpressing *ONAC066* showed improved tolerance to oxidative and drought stress and increased abscisic acid (ABA) sensitivity [26]. In soybean, the overexpression of *GmNAC20* enhances salt and freezing tolerance and accelerates the formation of *Arabidopsis* lateral roots, while the overexpression of *GmNAC11* only improves salt tolerance [29]. TaNAC30, a transcriptional activator in the wheat nucleus, was induced in a compatible wheat–Pst interaction and negatively regulated Pst resistance in wheat by restraining the expression of pathogenesis-related genes and the accumulation of hydrogen peroxide (H_2_O_2)_ [30]. Although this family was studied in numerous species, there are still few research studies on the role of NAC TFs in plant flowering. In addition, we have not elucidated the molecular mechanism of NAC TFs to perform those functions in *Cleistogenes songorica*.

*C. songorica* is an important forage and ecological grass widely distributed in desert areas of Northwest China, with an annual rainfall between 100 and 200 mm [31]. *C. songorica* is known for its extreme stress tolerance and dimorphic floret [32,33]. Overexpression of the *CsALDH* gene improves salt and drought resistance in transgenic *Arabidopsis thaliana* and alfalfa [34,35]. There are two different types of flower of chasmogamous (CH) and cleistogamous (CL) in different positions of *C. songorica*. There are many differences in the morphology of pollen between dimorphic florets, such as pollen germination aperture dimensions and exine ornamentation [36]. CL transgenic cultivars are a strategy to reduce environmental risks caused by transgenic plants. Clarifying the molecular mechanism of NAC transcription factors in response to abiotic stress and CL flowering in *C. songorica* could offer effective support for the genetic improvement of crops. However, notably, no *NAC* genes have been functionally characterized in *C. songorica*. In this research, we performed the genomewide identification, chromosome location, phylogenetic classification, gene structure, expression analysis and coexpression networks of the NAC TFs in *C. songorica*. Furthermore, CL flowering-related and abiotic stress-associated genes were also investigated.

## 2. Materials and Methods

### 2.1. Identification of NAC Genes in C. songorica

The genome sequences and RNA-seq data of *C. songorica* were obtained from Lanzhou University. The *Arabidopsis thaliana* and rice *NAC* gene sequences were obtained from the Phytozome database (https://phytozome.jgi.doe.gov/pz/portal.html) and were used to identify homologous peptides from the *C. songorica* genome by using the BLAST program. An online website (http://weizhongli-lab.org/cd-hit) was used to remove redundant genes with the default setting. NAC TFs which only contained the NAM domain (PF02365) were predicted using the Hidden Markov Model in the Pfam database.

### 2.2. Chromosome Localization, Gene Duplication and Syntenic Analysis

All *CsNAC* genes were labeled to *C. songorica* chromosomes using the Mapchart program (https://www.wur.nl/en/show/Mapchart.htm). OrthoMCL soft V5 was used to identify the duplicated genes with default setting. The syntenic relationships of *CsNAC* and *OsNAC* genes were illustrated with Tbtools (https://github.com/CJ-Chen/TBtools). The Ka, Ks and Ka/Ks values were calculated by the PAML yn00 NG model (http://abacus.gene.ucl.ac.uk/software/paml.html). Based on the rice λ value (6.5 × 10^−9^), the divergence time of *NAC* duplicated genes was calculated (T = Ks/2λ × 10^−6^ Mya).

### 2.3. Protein Properties, Conserved Motif, Gene Structure and Phylogenetic Analysis

The sequence length, molecular weight and isoelectric point of each NAC protein were analyzed by ExPasy (http://www.expasy.org/tools/). Orthologous genes in *Arabidopsis thaliana* of *CsNAC* genes were predicted using the BLASTP program at TAIR (https://www.arabidopsis.org). A neighbor-joining (NJ) phylogenetic tree was constructed using the MEGA7 program (1000 bootstrap replicates). The gene structure of *CsNAC* genes was visualized using the GSDS tool (http://gsds.cbi.pku.edu.cn/index.php). The MEME online website (http://meme.ebi.edu.au) was used to predict *CsNAC* gene conserved motifs.

### 2.4. Gene Expression Analysis in Various Tissues and under Multiple Stress Treatments

*C. songorica* was planted according to a previous report [37]. All seedlings were grown in a growing room at 26 ± 2 °C, under photosynthetically active radiation of 150 μmol m^−2^ s^−1^, a 16 h photoperiod and 65% relative humidity. Each pot contained 450 g of sand/vermiculite mixture, and the seedlings were watered with 100 mL Hoagland’s solution every 3 days. Nine week-aged plants were treated with heat (40 °C), cold (4 °C), low salt (50 mM NaCl), middle salt (100 mM NaCl), high salt (200 mM NaCl) and ABA (100 μmM ABA) for 24 h. Eleven week-aged plants were treated with low drought (LD, 6–10% soil water content) and high drought (HD, 1–3% soil water content) for 2 weeks and recovery 48 h after high drought. Shoots and roots were collected after treatment, frozen immediately in liquid nitrogen and stored at −80 °C. The expression levels (FPKM) of the *CsNAC* genes in various tissues (roots, leaves, seeds, cleistogamous (CL) flowers and chasmogamous (CH) flowers) and multiple stress treatments were obtained from RNA-seq data (unpublished data). The ln-transformed values for the FPKM of *CsNAC* genes were used for expression profile analysis at Omicshare (http://www.omicshare.com).

Total RNA was extracted from *C. songorica* shoots under four treatments (high temperature, low temperature, salt, drought stress) using the Sangon Biotech RNAiso reagent kit. The extracted RNA was removed underlying genomic DNA and then reverse-transcribed into first-stand cDNA using the TaKaRa reagent Kit. PerlPrimer v1.1.21 software was used to design specific primers based on the *CsNAC* gene sequences. The qRT-PCR reaction was performed according to the instructions of the 2 × TaqMan Fast qPCR Master Mix kit (Sangon Biotech, Shanghai, China). The correlative expression was calculated according to the ∆∆ Ct method. The expression quantity of *CsGADPH* gene was used as an internal control [38].

### 2.5. Coexpression Network Construction and Gene Annotation Analysis

The coexpression network of *CsNAC* genes was constructed using R package (WGCNA; weighted gene coexpression network analysis). Fifty-nine expression data sets were applied for the stress-related coexpression analysis, and 14 expression data sets were used for the CL flower development coexpression analysis. Coexpression networks were displayed by Cytoscape v 3.7.2. KOBAS 3.0 was used to annotate coexpressed genes.

## 3. Results and Discussion

### 3.1. Identification of NAC TFs in C. songorica

*C. songorica* is an important native perennial forage and ecological grass in Northwest China. In recent years, we performed whole-genome sequencing of *C. songorica* and conducted a large number of transcriptome sequences to mine its genetic resources. In this research, we identified 162 *NAC* genes in *C. songorica* genome. The *NAC* genes were named *CsNAC001* to *CsNAC162* (Table S1). The number of *NACs* identified in *C. songorica* was similar to rice (151) [39], soybean (152) [40] and *Vitis vinifera* (163) [41] and redundant to *Arabidopsis thaliana* (117) [39] and *Brachypodium distachyon* (101) [42]. Overall, the results indicated that the NAC TFs did not undergo special expansion in *C. songorica*. Furthermore, the isoelectric points (pIs), amino acid residues and molecular weights (Mws) were analyzed. The 162 CsNAC proteins varied from 157 (CsNAC004) to 856 (CsNAC076) amino acid residues with an average of 378.5 aa. The relative Mws ranged from 17.8 (CsNAC004) to 96.1 (CsNAC076) kDa with an average of 41.8 kDa, and the pIs ranged from 4.48 (CsNAC003) to 11.3 (CsNAC063) with 101 members showing isoelectric point (pI) ≤ 7 and the remainders showing pI > 7 (Table S1), suggesting that NAC TFs may act under a different physiological environment [10].

### 3.2. Phylogenetic Relationship and Evolutionary Analysis of CsNAC and OsNAC TFs

**Figure 1:**
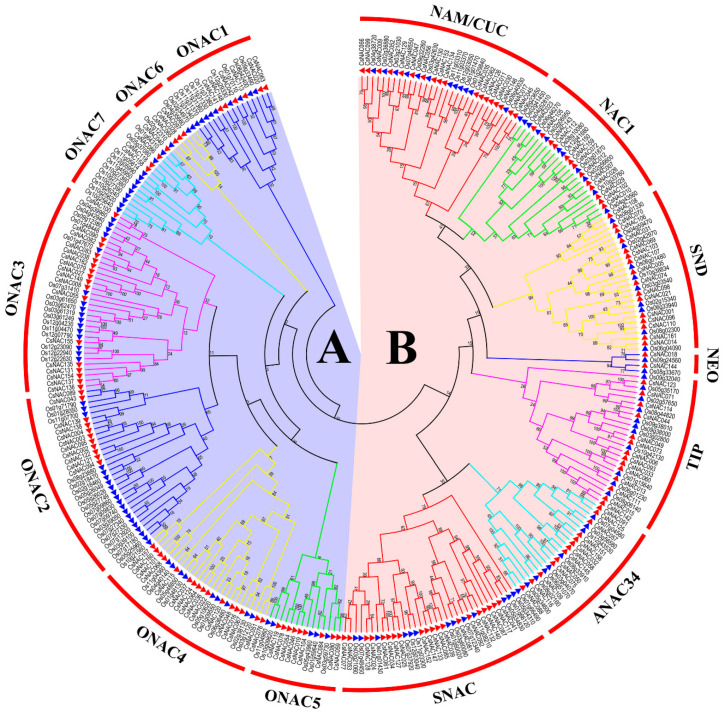
Phylogenetic tree of NAC transcription factors (TFs) in *C. songorica* and rice. The neighbor-joining (NJ) phylogenetic tree was constructed using the MEGA7 program with 1000 bootstrap replicates. CsNAC and OsNAC proteins were labeled by triangles with red and blue, respectively.

**Figure 2:**
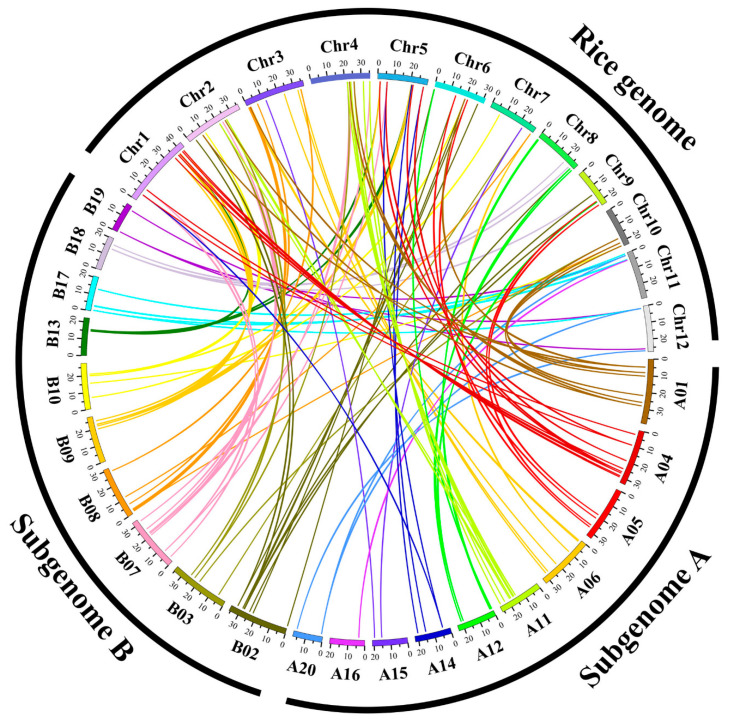
Schematic representations of syntentic relationships of *NAC* members between *C. songorica* and rice. Colored lines indicate *NAC* orthologous genes in the *C. songorica* and rice genomes. Subgenomes A and B represent 20 *C. songorica* chromosomes, while Chr1 to Chr12 represent rice. The chromosome number is marked below each chromosome.

To determine the evolutionary relationships between CsNAC and OsNAC proteins, a neighbor-joining phylogenetic tree was performed. As shown in Figure 1, CsNAC proteins were separated into two major groups—A and B (Figure 1). The result is consistent with a previous study [39]. Group A and B were separated into 7 subfamilies, respectively. Subfamily SNAC contained twenty CsNAC protein members, whereas only two CsNAC proteins were found in subfamily NEO (Figure 1). Each subfamily contains OsNAC and CsNAC proteins (Figure 1), which may indicate that no subfamily loss occurred in the NAC family after *C. songorica* and rice differentiation. The subfamily SNAC is related to the plant response to stress [43], and contains 13 OsNAC proteins and 20 CsNAC proteins (Figure 1), which indicates that the *NAC* gene from *C. songorica* is more closely related to stress than rice. Most subfamilies contain paralogous gene pairs, such as SNAC with 7 pairs, followed by subfamily ONAC4 with 6, but no paralogous gene pairs were found in NEO and ONAC1, and all of the paralogous gene pairs were from the same subfamily (Figure 1 and Figure 2).

**Figure 3:**
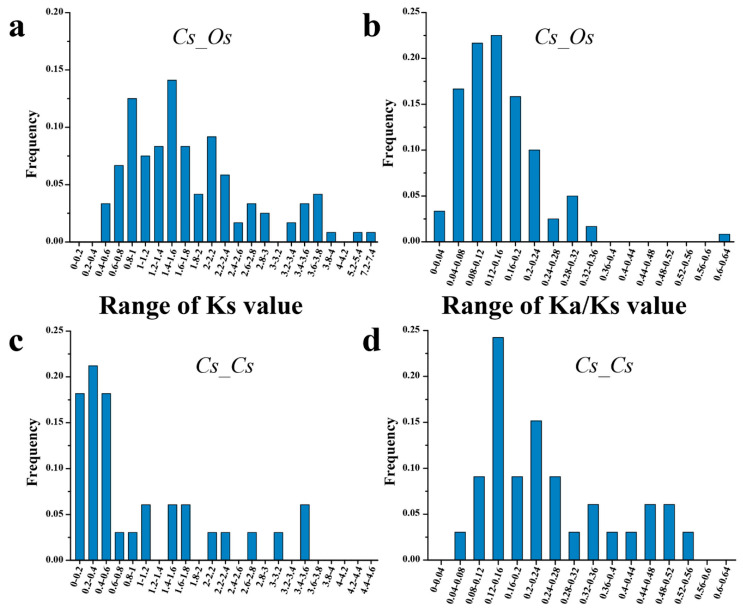
Ks and Ka/Ks value distributions of the NAC members in C. songorica and rice genomes. The distribution of Ks and Ka/Ks values of orthologous genes between C. songorica and rice (**a**,**b**) and paralogous genes in C. songorica (**c**,**d**).

Syntentic analysis was usually used to identify homologous genes and evolutionary relationships between genes [44]. To explore the evolutionary relationships between rice and *C. songorica* NAC family, we performed a comparative syntenic analysis and found 68 *OsNACs* and 93 *CsNACs* as orthologs (Figure 2). All orthologous gene pairs come from the same subfamily (Figure 1), which provides potential support for the accuracy of the phylogenetic tree. Among orthologous genes, twenty-eight pairs of orthologous genes were one-to-one, including *CsNAC013* - *LOC_Os06g01230.1* and *CsNAC156* - *LOC_Os12g43530.1*. The results indicated these *NACs* evolved from the common ancestor of rice and *C. songorica*. We further analyzed the evolutionary pressure between orthologous genes (Cs-Os). The Ks values peaked at 0.6–1.8 between *C. songorica* and rice (Figure 3a). The Ka/Ks ratios were primarily distributed at 0–0.36, with the maximum value being observed in the *CsNAC059 - LOC_Os03g59730.1* pair (Ka/Ks = 0.62) (Figure 3b: Table S2), indicating that they undergo purifying selection during biological evolution between two species [37].

### 3.3. CsNAC Gene Structures and Conserved Motifs

**Figure 4:**
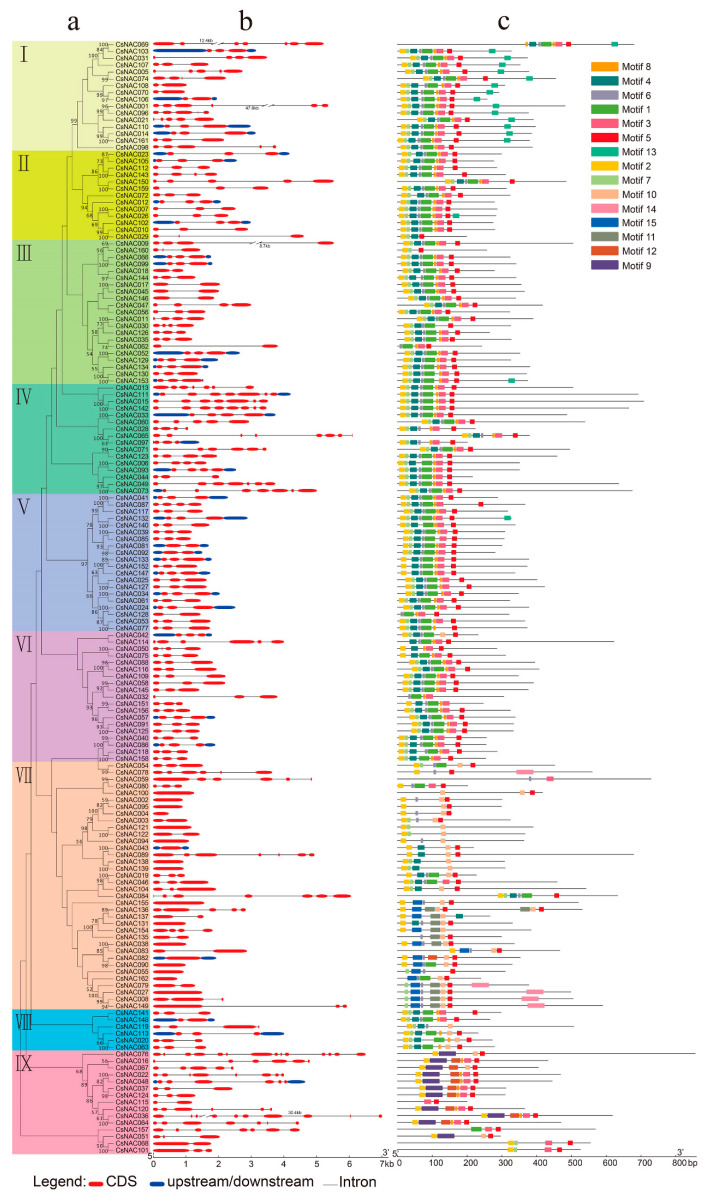
Phylogenetic relationships, gene structure and putative conserved domain distributions of the CsNAC family. (**a**) The phylogenetic tree was constructed with CsNAC protein sequences using MEGA 7 software. The cluster details are indicated in multiple colors. (**b**) Exon–intron structure is indicated by red rectangles and black lines, respectively. (**c**) Multiple colored boxes represent different motifs. The motif sequence information is provided in Figure S1.

A phylogenetic tree was performed with CsNAC protein sequences, which clustered the NAC proteins into 9 subgroups (Figure 4a). Gene structural diversity is a possible mechanism for the evolution of gene families [45]. To explore the structural diversity of *CsNACs*, we compared the intron/exon structure of each *NAC* gene in *C. songorica*. Generally, *CsNAC* genes with high bootstrap supported in the same subgroups that shared similar intron/exon structures in points of exon number and length (Figure 4b). For example, the *CsNAC* genes in subgroups III, V and VIII included 2 to 4 exons and those in subgroup II contained 3 to 5 exons. Inversely, subgroups VII and IX showed a large difference with exon numbers ranging from 1–14 (Figure 4a,b). These results indicated that the II, III, V and VIII subgroups are more conserved, while VII and IX could be related to the evolution of the CsNAC family.

**Figure 5:**
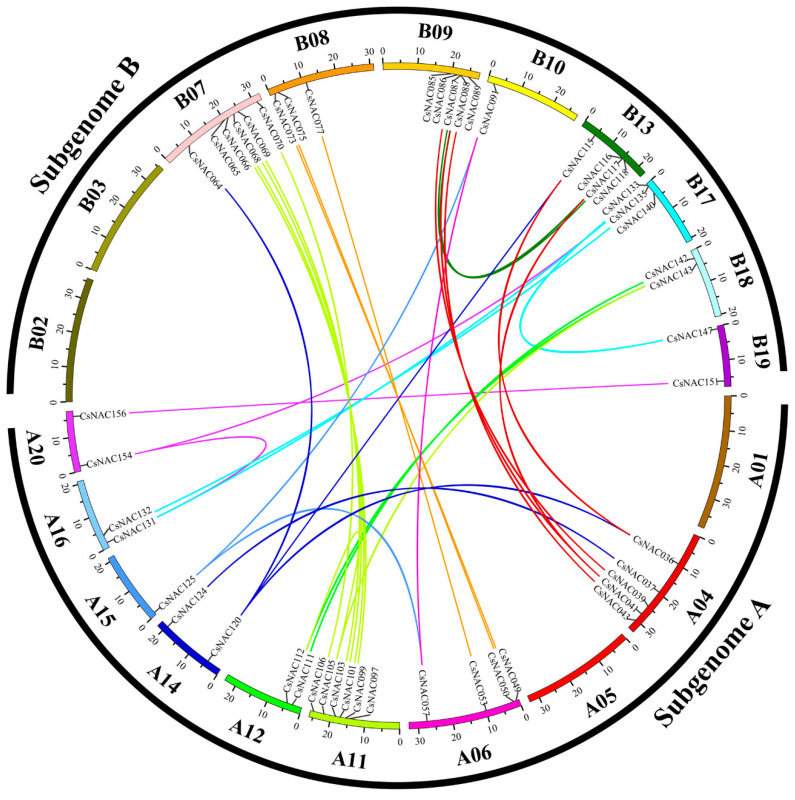
Distribution of the *NAC* duplicated genes in *C. songorica.* The colored lines within the chromosomes represent the syntentic relationships in *C. songorica NAC* members.

To explore intron gain or loss information about the NAC family in *C. songorica*, we further performed the intron/exon structure of *CsNAC* paralogous gene pairs. Among 33 paralogous pairs, 25 pairs displayed similar intron/exon structure in points of either intron numbers or gene length (Figure 4b and Figure 5). Sixteen paralogous pairs displayed a highly conserved gene structure with the same exon numbers (Figure 4b). However, others showed a certain degree of difference. Nine *CsNAC* genes of the 33 paralogous pairs possessed one more exons than and a similar length to their paralogous genes (Figure 4b and Figure 5). We speculate that the differences may be due to single intron loss or gain that occurred during the structural evolution of *NAC* paralogs.

To further reveal the functional diversification of NAC transcription factors in *C. songorica*, conserved motifs were analyzed using the MEME program, and 15 conserved motifs were predicted. As expected, members with high bootstrap support in the phylogenetic tree possessed highly similar motif compositions (Figure 4c). Motifs 2, 4, 3, and 5, respectively, represent the A, B, D and E subdomains of the NAM domain, while 6 and 1 represent subdomain C. Subdomain A is related to the formation of functional dimers. The highly conserved positively charged subdomains C and D bind to DNA, while subdomains B and E are involved in the functional diversity of the NAC TFs [8]. In fact, NAC TFs can regulate the expression level of downstream genes through complex interactions between the DB domain, NARD and activation domain [46]. Some CsNAC proteins (such as CsNAC043, CsNAC037, CsNAC101) have only partial motif composition of the NAM domain, and we call this NAC-like protein (Figure 4c) [43]. Furthermore, motifs 11, 14 and 15 were unique to subgroup VII, motifs 9 and 12 were only found in subgroup IX, motif 13 was present mainly in subgroup I, and motif 10 was mainly in subgroups VII, VIII, IX (Figure 4a,c). The specific structure of these subgroups may confer their potential specific functions [47]. A total of 66.7% (22/33) *NAC* paralogous gene pairs possessed the same motif compositions as one another, indicative of functional similarities. Inversely, special motifs were observed for the remaining 11 pairs of NACs; for example, CsNAC053 and CsNAC077 are different in motif 3 (Figure 4c and Figure 5).

### 3.4. Genomic Locations and Duplication of the NAC Genes in C. songorica

**Figure 6:**
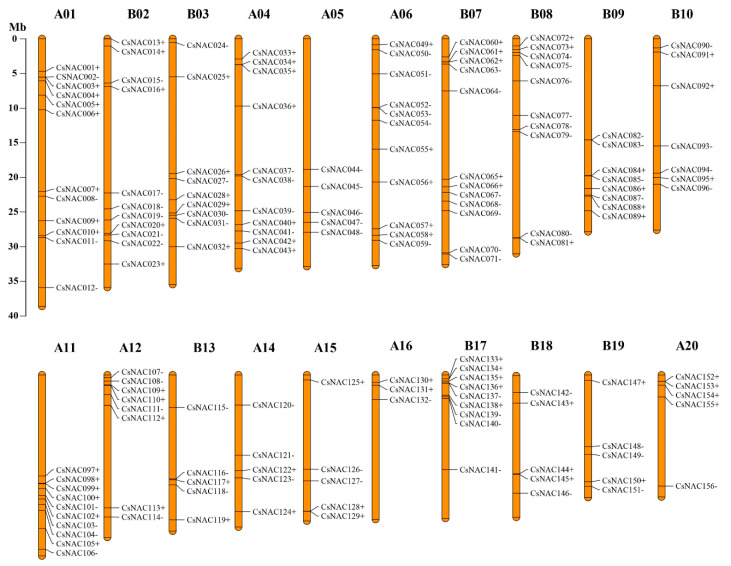
Chromosomal location of *C. songorica NAC* genes. The chromosome number is above each chromosome. The scale represents chromosome length.

As shown in Figure 6, a total of 156 (96.3%) *CsNACs* were unequally distributed on 20 chromosomes of *C. songorica*. Chromosomes A01 and B07 had the most *CsNAC* genes (21, ~25.93%), whereas there were only 3 genes (1.9%) in chromosome A16 (Figure 4). Subgenomes A and B possessed 75 (42.3%) and 81 (50%) *CsNAC* genes, respectively (Figure 6).

Gene duplication is a mechanism for gene family expansion and new gene generation. In rice, gene family expansion is primarily involved in segmental duplication, tandem duplication, and transposition events [48]. To find *NAC* duplicated genes in *C. songorica* genome, the syntentic analysis was carried out using OrthoMCL software. In this research, 33 pairs of duplicated genes were found in 162 *CsNAC* genes (Figure 5). These duplicated genes were the most common on chromosomes B07 and A11, whereas there were no duplicated gene pairs on chromosomes A01, B02, B03 and A05 (Figure 5). All *CsNAC* paralogous gene pairs existed on different chromosomes, and no tandem duplication genes were found. A total of 72.7% *CsNAC* duplicated gene was found between subgenome A and B in *C. songorica*, which was the same as the bZIP family (72.5%). In addition, a previous study indicated that *C. songorica* experienced a whole-genome duplication event during biological evolution [37]. These results suggested that whole-genome duplication events resulted in the expansion of the NAC family in *C. songorica*. *NAC* genes in pear undergo purification selection and positive selection while only undergoing purification selection in cotton [49,50]. In this study, we analyzed the evolutionary pressure between *CsNAC* duplicated genes (Cs-Cs) and found that the Ka/Ks ratios of all *NAC* duplicated genes were less than 1, indicating that *NAC* genes underwent purifying selection in the *C. songorica* genome (Figure 3d; Table S3). In addition, the frequency distributions of Ks value peaked at 0–0.6, and their divergence times were 2.0–272.4 MYA (Figure 3c; Table S3).

### 3.5. Expression Profiles and Coexpression Network Analysis of CL Flowering Related CsNAC Genes 

**Figure 7:**
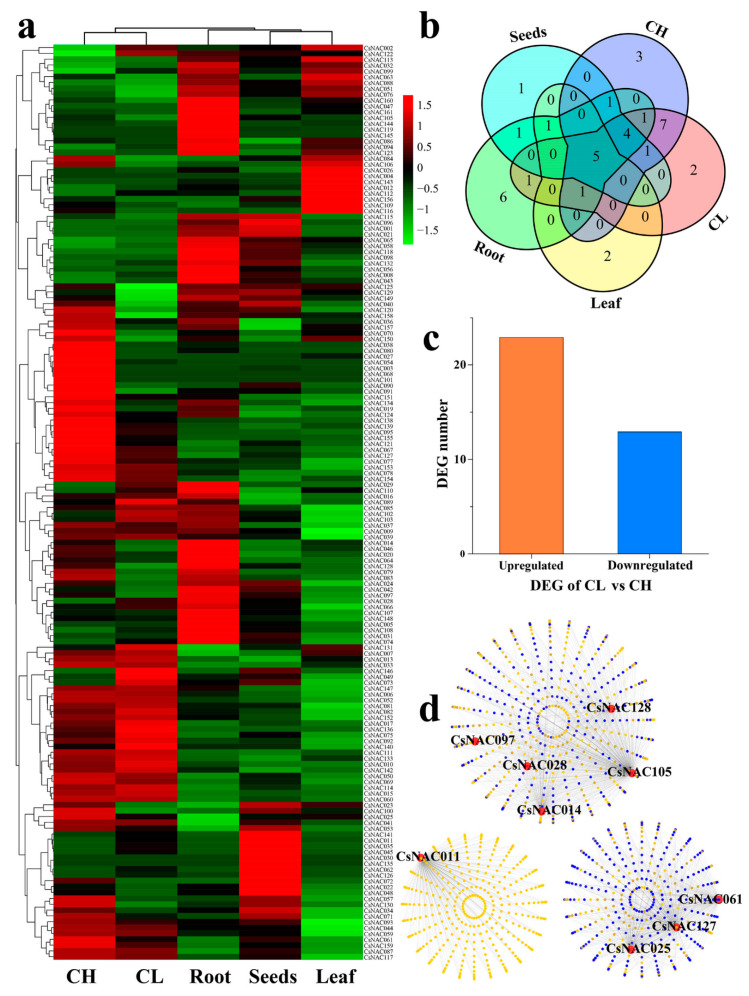
Expression profiles and coexpression network of CsNAC TFs during dimorphic floret development. (**a**) Hierarchical clustering of *CsNACs* in multiple organs based on transcriptome data. (**b**) The highly expressed *CsNACs* (FPKM ≥ 20) in multiple organs was marked by the overlapping petal circle. (**c**) The number of up-regulated and down-regulated *CsNAC* differentially expressed genes (DEGs) in dimorphic florets development. (**d**) Coexpression network of *CsNAC* DEGs between cleistogamous (CL) and chasmogamous (CH) flowers. The light yellow points represent individual genes. The blue points represent overlapping genes. The dark yellow points represent yellow points overlying blue points.

We performed the expression profiles of *CsNAC* genes in multiple organs, including the seeds, leaves, roots, chasmogamous (CH) and cleistogamous (CL) flowers (Figure 7a). A total of 112 (69.1%) *CsNAC* genes had expression in at least one organ (FPKM≥1), and 44 (27.2%) *CsNACs* had expression in all organs (FPKM≥1). Furthermore, 37 (22.8%) *CsNACs* highly expressed in at least one organ (FPKM≥20) (Figure 7a; Table S4). Five *CsNACs* had high expression in all organs, including *CsNAC041*, *CsNAC081*, *CsNAC087 CsNAC123* and *CsNAC148* (Figure 7b; Table S4). Strikingly, 12 *CsNACs* had high expression in dimorphic florets but not in roots and leaves; 8 *CsNACs* had high expression in roots but not in dimorphic florets (Figure 7a; Table S4).

There are approximately 700 cleistogamy plants in the world, such as *Pisum sativum* and *Impatiens balsamina*. Cleistogamy was separated into three types: complete cleistogamy, dimorphic cleistogamy and induced cleistogamy [51]. Compared with chasmogamous, cleistogamy can produce seeds by self-pollination to maintain the population’s reproduction under extreme environments. *C. songorica* is a dimorphic cleistogamy plant that produces open (chasmogamous) flowers at the apical meristem and closed (cleistogamous) flowers in the sheath. CL in the sheath has a significant impact on the seed yield of *C. songorica* [36]. Thirty-six *CsNAC* genes had differential expression |log_2_ (fold change) ≥ 1| between dimorphic florets, including 23 upregulated and 13 downregulated *CsNACs* (Figure 7c; Table S4). GO annotation indicated that these *CsNACs* were related to the biosynthetic process, response to stimulus, organ development, floral whorl development, anther development and dehiscence (Table S5). The NAM subfamily is involved in plant development. In this subfamily, *EPHEM-ERAL1* (*EPH1*), positively regulates PCD (programmed cell death) during petal senescence in Japanese morning glory (*Ipomoea*) [52]. The sly-miR164-regulated *SlNAM2* genes play key roles in tomato floral-boundary specification [53]. The *CsNAC011*, *CsNAC099*, *CsNAC130*, *CsNAC134* and *CsNAC146* genes in the NAM subfamily showed differential expression between CL and CH flowers, and we speculate that they may also play vital roles in the cleistogamy of *C. songorica*.

Genes with similar expression patterns may be coregulated, functionally related, or in the same pathway. WGCNA can predict the regulatory relationship between genes and the function of unknown genes based on expression data, providing important clues for the study of unknown genes [54]. Nine dimorphic floret-related *CsNAC* genes (*CsNAC011*, *CsNAC014*, *CsNAC025*, *CsNAC028*, *CsNAC061*, *CsNAC097*, *CsNAC105*, *CsNAC127* and *CsNAC128*) were used for coexpression analysis using WGCNA. A total of 2372 coexpressed genes were identified, and 917 coexpressed genes showed overlap with these 9 genes (Figure 7d; Table S6). GO annotation indicated that these *CsNACs* were related to the developmental process, reproductive process, flower development, pollen exine formation and response to stress (Table S6). The result indicated these *CsNACs* can be involved in dimorphic floret differentiation by regulating flower development and reproductive processes.

### 3.6. Expression Profiles and Coexpression Network Analysis of Abiotic Stress-Related CsNAC Genes

NAC transcription factors participate in plant response to various abiotic stresses. Overexpression of *SNAC1* enhances transgenic rice drought resistance in the field during the reproductive stage [55]. The transgenic alfalfa overexpressing *MfNAC3* had better cold resistance, and the expression levels of cold response genes *MtCBFs* and *MtCASs* were significantly increased [27]. Although many stress-related NAC TFs have been studied in various species, there is still no research on *C. songorica*.

**Figure 8:**
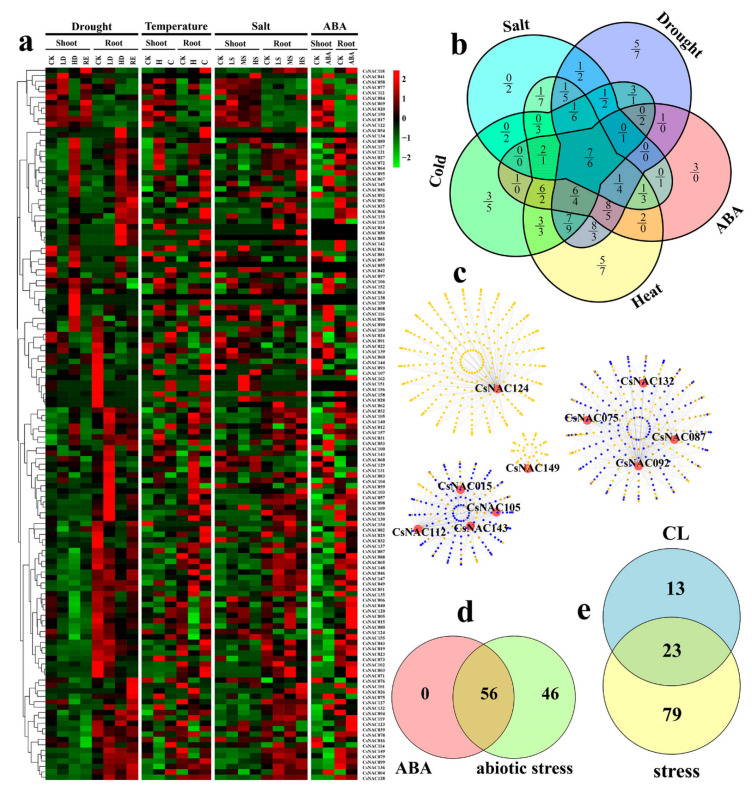
Expression profiles and coexpression network analysis of abiotic stress-associated *CsNAC* genes. (**a**) The expression profiles of *CsNAC* genes under five treatments (drought, heat, cold, salt and ABA). (**b**) The *CsNAC* DEGs under five treatments (high drought, heat, cold, high salt and ABA) were marked by the overlapping petal circle. The numbers above and below the line represent the *CsNAC* DEGs in shoots and roots, respectively. (**c**) Coexpression network of *CsNAC* genes with DEGs under abiotic stress. The light yellow points represent individual genes. The blue points represent overlapping genes. The dark yellow points represent yellow points overlying blue points. (**d**) The *CsNAC* DEGs under abiotic stress and ABA treatment was marked by the overlapping circle. (**e**) The *CsNAC* abiotic stress-associated and CL flowering-related genes were marked by the overlapping circle. CK: control; LD: low drought; HD: high drought; RE: recovery H: heat; C: cold; LS: low salt; MS: middle salt; HS: high salt.

To explore the role of *CsNAC* genes in abiotic stress, we performed the expression profile of *CsNAC* genes in multiple abiotic stress-related treatments, including drought, high temperature, low temperature, salt and ABA. Eighty-one (50.0%) and 96 (59.3%) *CsNACs* had expression under at least one treatment (FPKM ≥1) in shoots and roots (Figure 8a; Table S7), respectively. Moreover, 77 (47.5%) and 93 (57.4%) *CsNACs* showed differential expression |log_2_ (fold change) ≥1| under at least one treatment in shoots and roots, respectively (Figure 8b).

A total of 59, 40, 16, 50 and 38 *CsNACs* had differential expression under high temperature, low temperature, salt, drought stress and ABA treatments in shoots, respectively. Sixty-eight, 47, 45, 57 and 29 *CsNACs* had differential expression under the heat, cold, salt, drought stress and ABA treatments in roots, respectively (Figure 8b). Interestingly, 7 and 6 of DEGs (differentially expressed genes) overlapped under five treatments in shoots and roots, respectively (Figure 8b). In total, 102 *CsNACs* had differential expression under five treatments in two organs (Figure 8d; Table S7). The results showed that *CsNAC* genes participated in regulating abiotic stress. A Venn diagram showed that more *CsNACs* responded to drought and high temperature than responded to low temperature and salt stress (Figure 8b), indicating that *CsNAC* genes might play crucial roles in the response to drought and high temperature in *C. songorica*. These finding may be observed because *C. songorica* usually grows in desert areas. The stress-related *CsNACs* were primarily distributed in subfamilies SNAC, ONAC4, ONAC3, NAC2 and ONAC1. Eighty percent of SNAC genes showed differential expression in shoots and roots under five treatments (Figure 1; Table S7), showing that the SNAC subfamily is closely related to abiotic stress. The result was consistent with the findings of a previous study [43]. We further analyzed the relationship between ABA-responsive and abiotic stress-responsive *CsNAC* genes. Fifty-six *CsNACs* responded to ABA and abiotic stress simultaneously, while 46 *CsNACs* specifically responded to abiotic stress (Figure 8d). The result indicated that more ABA-dependent *CsNAC* genes participated in the response to abiotic stress than ABA-independent.

Ten stress-related *CsNAC* genes were used for the coexpression network analysis. There were 1582 coexpression genes in the coexpression network, of which 545 were overlapping coexpression genes (Figure 8c; Table S8). Moreover, we performed the GO annotation of 1582 coexpression genes, and found that some of these genes were related to stress-responsive GO terms, for example: the response to stress, response to hormone, defense response and oxidation-reduction process (Table S8). Strikingly, some coexpression genes were related to flower development, including reproductive process, flower development and photoperiodism flowering (Table S8).

We ulteriorly found that 23 *CsNAC* genes had differential expression in dimorphic florets and under abiotic stress (Figure 8e). Sang-Gyu Kim (2007) found that NTL8, an NAC transcription factor, regulates salt-responsive flowering via FLOWERING LOCUS T in *Arabidopsis* [56]. Furthermore, a previous study revealed that DEGs between CH and CL flowers were related to defense responses [57]. The results suggested that CL flowers could be induced by environment, while NAC transcription factors may be involved in related regulatory processes.

**Figure 9:**
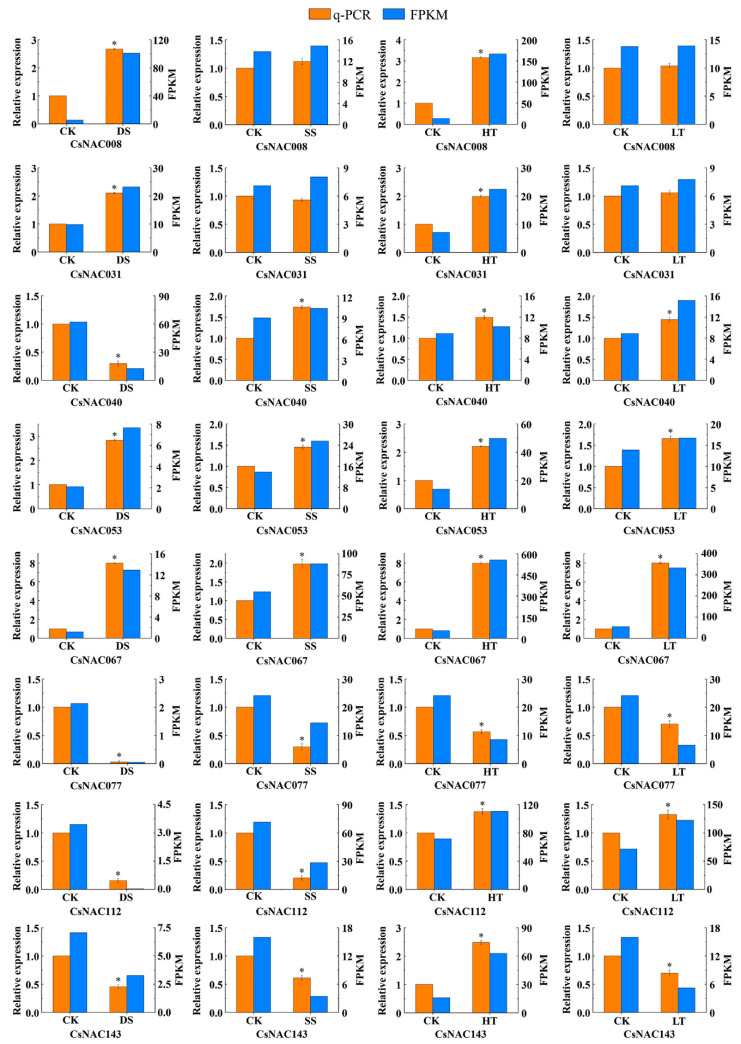
The expression of selected *CsNAC* genes under abiotic stress in shoots by qRT-PCR. DS: drought stress; SS: salt stress; HT: high temperature; LT: low temperature. Bars represent means ± standard error (*n* = 3). Asterisks indicate relative expression significant differences under abiotic stress (*p* < 0.05). The *CsGAPDH* was used as the internal control.

The expression patterns of some stress-related *CsNAC* genes were verified by qRT-PCR under drought (2% soil water content), salt (100 mM NaCl; 24 h), high temperature (40 °C; 24 h) and low temperature (4 °C; 24 h). The qRT-PCR results were in keeping with the transcriptome data Figure 9 indicating that these 8 *CsNACs* were involved in four abiotic stresses. For example, *CsNAC077*, *CsNAC112* and *CsNAC143* were downregulated under drought and salt stress. *CsNAC053* and *CsNAC067* were upregulated under heat and cold stress.

## 4. Conclusions

One hundred and sixty-two *NAC* genes from the *C. songorica* genome were identified, and their basic classification and evolutionary characteristics were analyzed. A genomewide analysis including gene structure, chromosomal location, synteny, expression profiles, coexpression network and GO annotation of the NAC family in *C. songorica* was first performed. We highlighted the role of CsNAC transcription factors in response to abiotic stress and CL flower development. These results may be helpful to the follow-up research on the role of the *CsNAC* genes in dimorphic floret development and stress response.

## Supplementary Materials

The following are available online at https://www.mdpi.com/2073-4425/11/8/927/s1. Table S1: Characteristics of *CsNAC* genes in *C. songorica*. Table S2: Synteny blocks of *NAC* genes between *C. songorica* and rice genome. Table S3: Synteny blocks of *CsNAC* genes within *C. songorica* genome. Table S4: Expression pattern of *CsNAC* genes in different organ. Table S5: GO annotation of DEG between CH and CL flowers. Table S6: Gene list and GO annotation of coexpression genes with CL flower development genes. Table S7: The transcriptome data of *CsNAC* genes in shoot and root under abiotic stress. Table S8: Gene list and GO annotation analysis of coexpression genes with abiotic stress related genes. Table S9: Primer list for gene-specific primers. Figure S1: Overview of 15 conserved motifs identified through MEME analysis. The raw data of the *C. songorica* whole-genome sequence were submitted to NCBI, number: PRJNA634005; the genome assembly sequence was submitted to the National Genomics Data Center (NGDC), number: PRJCA002752.
