# Supplementary figures and images for: Genome-Wide Analysis of the Role of NAC Family in Flower Development and Abiotic Stress Responses in Cleistogenes songorica
Source: Genes (Basel). 2020 Aug 12;11(8):927. doi: 10.3390/genes11080927 (PMC7464430; doi:10.3390/genes11080927)

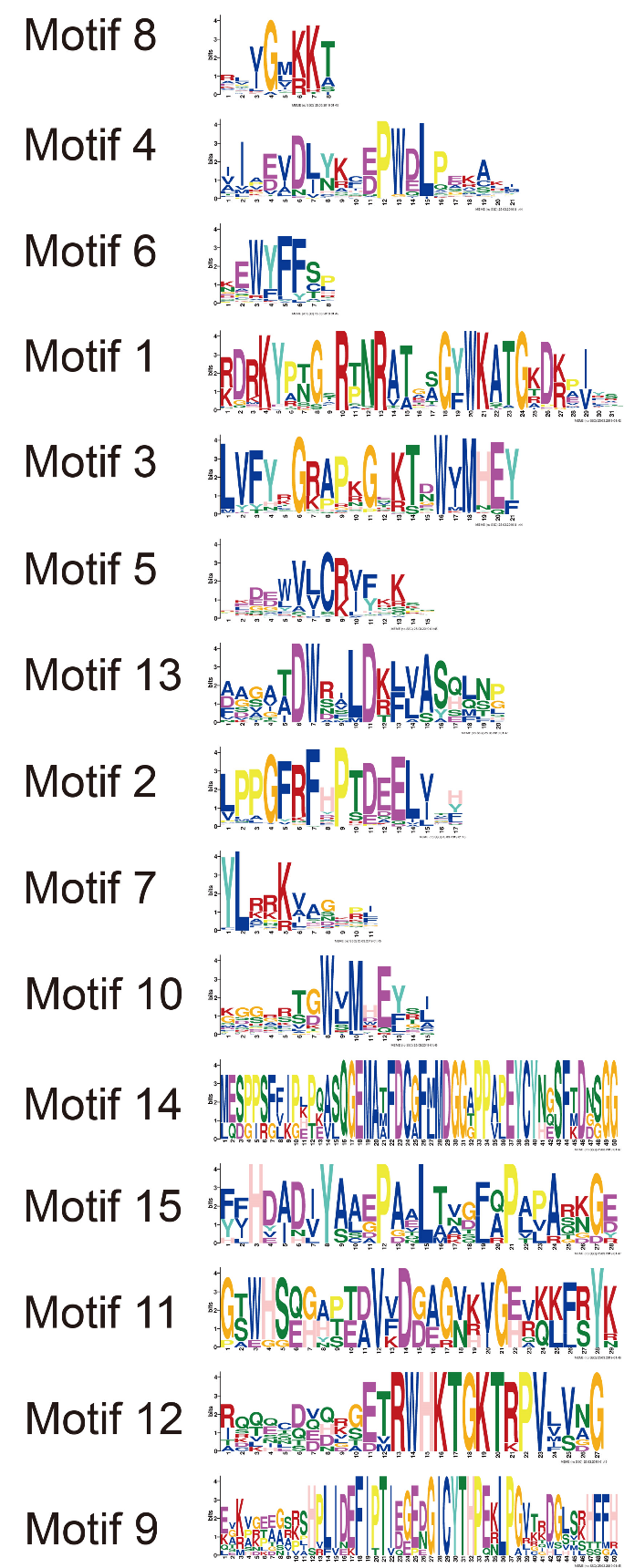


**Figure S1** Overview of 15 conserved motifs identified through MEME analysis

Supplement: Supplementary file 1 [file genes-11-00927-s001.zip › Supplementary materials/Figure S1.docx]
